# Cotton Cellulose-Derived Hydrogel and Electrospun Fiber as Alternative Material for Wound Dressing Application

**DOI:** 10.1155/2022/2502658

**Published:** 2022-03-07

**Authors:** Supidcha Jirawitchalert, Samon Mitaim, Ching-Yi Chen, Nisa Patikarnmonthon

**Affiliations:** ^1^Department of Biotechnology, Faculty of Science, Mahidol University, 272 Rama VI Rd., Phayathai, Ratchathewi, Bangkok 10400, Thailand; ^2^Department of Chemical Engineering, National Chung Cheng University, Chia-Yi County 62102, Taiwan; ^3^Mahidol University-Osaka University: Collaborative Research Center for Bioscience and Biotechnology, Faculty of Science, Mahidol University, 272 Rama VI Rd., Phayathai, Ratchathewi, Bangkok 10400, Thailand

## Abstract

Cotton has been recognized as a useful biomaterial over decades, and it has been widely applied in the textile industry. However, a large amount of cotton waste is generated during the manufacturing processes, but it has been considered as a low-value product. With high content of cellulose remaining in cotton waste, our study focuses on transforming cotton cellulose into a valuable product. Cellulose was extracted from cotton waste and modified into two main materials for wound dressing application: hydrogel-based water absorbent materials and electrospun composite nanofibers. In order to enhance the water absorption, carboxymethyl cellulose (CMC), the modified cellulose with functional group prone to interact with water molecules, has been developed in this study. The hydrogel-based CMC was created by using the chemical cross-linking reaction of epichlorohydrin (ECH). The hydrogel demonstrated the swelling and reswelling ability by 1718 ± 137% and 97.95 ± 9.76%, respectively. Meanwhile, cellulose/PEG in trifluoroacetic acid (TFA) was successfully fabricated as nonwoven composite by a conventional electrospinning technique. The fabrics provided highly appropriated properties as wound dressing, including the following: water absorption was up to 1300 times and water vapor permeability controlled in the range of 2163–2285 g·m^−2^·day^−1^. This showed the preliminary information for recovering cotton waste into valuable products.

## 1. Introduction

Wound, injury, or disruption of epithelium of skin or mucosa caused by the physical, chemical, mechanical, and/or thermal damages can be categorized into two types according to duration of healing: acute and chronic wounds [1, 2]. Acute wounds are normally expected to heal within 8–12 weeks [3], whereas chronic wounds fail to progress healing within normal healing time [4]. The wound healing is a biological process associated with the cytokines that promote the healing process by inducing the cell membrane repair, preventing the dehydration, and enhancing the inflammation. The healing processes are also affected by various factors such as unsuitable or deficient nutrition, amount of protein, vitamin, mineral, overall health, age, and individual diseases.

In order to provide protection to the wound from infection and allow healing process to occur, wound dressing is required. In general, the wound dressing must be sterile, nontoxic, and nonallergic. Apart from that, the ideal wound dressing should offer other additional properties for enhancing the effectiveness of the wound healing process, such as controlling of temperature, moisture, and gas-fluid exchange, antibacterial activity, or even ability to promote cell proliferation [5, 6]. Several materials were applied as wound dressers such as clay tablets (2500 BC). Traditional wound dressing appeared with basic materials to be composed as adhesive and nonadhesive bandages, as well as woven and nonwoven bandages. The main purposes were curing wound, absorbing exudate, and preventing the contamination. The important development occurred during the 19th century due to the introduction of antibiotics. The modern wound dressing was established; the current approach for wound dressing is based on foams [7], films [8], hydrogel [9], and nanofibrous mats [10], with natural polymers such as alginate, chitosan, and bacterial cellulose or synthetic polymers such as polyurethane.

Cellulose is one of the most abundant natural polymers found in plant cell walls; its function is to provide rigid structure to plant cells. It is the most popular source of cellulose as it is composed of up to 96.5% of cellulose [11]. According to its accessibility, being environmentally friendly, biocompatibility, and being an economically affordable raw material, cotton cellulose is one of the most commonly used organic polymers in several industries, for instance, textile industries, woven into clothes, socks, towels, and other nontextile materials such as tents and coffee paper [12]. Large amounts of cotton waste, up to 10 tons per month, are unrecoverable in the manufacturing process and turned into low-valued products such as rugs, oil absorption material, or substrate for mushroom cultivation. The attempt of cotton waste recovery has been done to reduce the number of cotton wastes by turning it into high value material. Cotton waste-based cellulose has already been modified as carboxymethyl cellulose with ability of superabsorbent material aiming for utilization as diapers [13, 14]. Cellulose-based superabsorbent has been developed based on hydrogel formation in presence of epichlorohydrin (ECH), a base-catalyzed cross-linking agent that acts as an etherification agent toward hydroxyl groups [15, 16]. By using a cross-linker, the network structure can be formed by linking the polymer chains to each other [17]. The gelation behavior is involved with chemical cross-linking, as well as hydrogen bonds between hydroxyl groups of cellulose, and solvent as intramolecular hydrogen bonding or intermolecular bonding. These cause electrostatic interaction between molecules and lead to the ability to absorb water [18]. The physical cross-linking can also create irreversible gelation from heating, which leads to self-assembled chain entanglements of the cellulose backbone [17]. According to the previous study [15], ECH can be used as a cross-linker for cellulose and cellulose derivative, carboxymethyl cellulose (CMC) in NaOH aqueous solution, by forming diether bond between hydroxyl groups of CMC and ECH. CMC can enhance the pore size of the hydrogel from the electrostatic repulsion of carboxylate anion (COO-), whereas the cellulose provides support for maintaining the structure of the gel due to its strong backbone which might be able to improve the swelling ability and property of the hydrogel.

Electrospinning is a recent general technique used for nanofiber fabrication which provided the imitated environment as extracellular matrix (ECM) [12]. Nanofiber can be formulated by spraying the polymer solution or melts incorporated with high-voltage electricity [19]. Electrospun nanofiber provides significant microbial barrier by arranging high surface area-to-volume ratio and high porosity [20], as well as ability to incorporate functional compound to enhance its property. Nanofiber composites can be processed by synthetic and natural polymers. Importantly, bio-based and environmentally friendly materials are considered. Thus, cellulose and its derivatives become noticeable polymers for electrospun composites [21].

In this study, cellulose purified from the cotton waste of textile company was used as a main polymer to prepare cellulose/CMC hydrogel and cellulose/polyethylene glycol (PEG) electrospun composite nanofiber for further combination as wound dressing materials. The improvement of absorbency of cellulose/CMC hydrogel was studied. Carboxymethyl cellulose was synthesized and tested to identify the suitable condition to enhance the swelling ability. Nonwoven fabrics of cellulose and PEG were also prepared by electrospinning technique. Methylene blue (MB), a member of the phenothiazinium family, is used as a model drug (photosensitizer) doped into the composite nanofibers. The MB is chosen due to its high quantum yield (Φ_*T*_≈0.52), long excitation wavelength, high solubility in aqueous media, and low cost compared to other photosensitizers in clinical use for antibacterial photodynamic therapy (Photodiagn. Photodyn. Ther. 2005, 2, 175–191). Then, the significant properties of electrospun as wound dressing were investigated.

## 2. Materials and Methods

### 2.1. Materials

Cotton waste was obtained from Erawan Textile Co., Ltd., Thailand. Sodium hydroxide was purchased from Merck, Germany. Urea and nitric acid were received from VWR Chemicals BDH. ECH and trifluoroacetic acid (TFA) were obtained from Alfa Aesar®. Methanol, sulfuric acid, isopropanol, acetic acid, ethanol, and acetone were purchased from RCI Labscan Limited. Sodium monochloroacetate (SMCA), methylene blue (MB), sodium carboxymethyl cellulose (CMC) (Mw ∼250,000, degree of substitution 0.7), sodium dodecyl sulfate (SDS), and Bovine Serum Albumin (BSA) were received from Sigma-Aldrich Co. Poly (ethylene oxide) (PEG) (Mn = 300,000) was purchased from ACROS Organics™.

### 2.2. Pretreatment and Bleaching of Cotton Waste

The cotton waste received was a mixture of cotton fiber, dust, small rubbish, and synthetic fiber. Visible impurities were removed prior to pretreatment and bleaching processes based on previously described protocol with some modification [22]. Sample fiber was cut with a length of 2-3 cm; 4 g of sample was treated with 20% (w/v) sodium hydroxide by stirring at 40°C for 2 hours. The sample was washed with water and then soaked with 200 mL of 6% (w/v) sodium hypochlorite for 16 hours for bleaching. The bleached sample was washed and left in the oven at 80°C until it was completely dried. 200 mL of 2% (v/v) sulfuric acid was added to the dried sample and then the suspended sample was placed into an autoclave to enhance the acid hydrolysis at 121°C, 15 psi, for 15 minutes. The sample was filtered through Whatman No.4 filter paper and air-dried at room temperature. At this step, the sample was changed into white powder form and considered as a pretreated cellulose for use in further steps.

### 2.3. CMC Synthesis

The method was modified according to the previous study [23]. The alkalization was started by adding 1 g of pretreated cellulose into 20 mL of 75% (v/v) isopropanol solution. Then, 2 mL of 20% (w/v) of NaOH solution was added dropwise into the sample and stirred at room temperature for 2 hours. Various amounts of sodium monochloroacetate (SMCA) ranging from 1.2 to 4.8 g (as shown in Table 1) were added to the mixture to start the etherification and the mixture was continuously stirred at 40–50°C for 3 hours. The cellulose sample was collected through filter paper and resuspended with 60 mL of methanol and then stirred further for 40 minutes at room temperature. Diluted acetic acid was used for neutralizing pH of the sample. Then the sample was collected by filtration and washed with 60 mL of methanol. The sample was left in the oven at 60°C for 3 hours or until it was completely dried.

### 2.4. Fourier Transform Infrared (FTIR) Spectroscopy

FTIR was used to measure the absorption or emission at infrared spectrum, in order to confirm the change of the functional groups and structural analysis of the samples. 1 g of pretreated cellulose and CMC samples were placed on the sample holder and dispersed to a thin layer. FTIR spectra were recorded on the FTIR system spectrometer (Bruker Hong Kong Limited, model ALPHA) using KBr pellets. FTIR spectroscopy was performed at a wavenumber range of 4000–500 cm^−1^.

### 2.5. Degree of Substitution of CMC

The degree of substitution of the carboxyl group onto the cellulose was determined by back titration method according to the previous report [22]. 1 g of CMC was added to 50 mL of 95% ethanol prior to addition of 5 mL of 2 M nitric acid. The mixture was stirred at room temperature for 10 minutes and then boiled for 5 minutes and further stirred for 20 minutes at room temperature. The sample was collected by filtration and washed with 100 mL of 95% ethanol before being placed in the oven at 90°C for 3 hours for drying. Then back titration was preceded by dissolving dried sample in 100 mL of distilled water and then adding 25 mL of 0.5 N NaOH. Then the sample was boiled for 20 minutes. 0.3 N HCl was used for titration, while the sample was hot. The amount of HCl used in titration step was measured and used to calculate the degree of substitution with the following equation:

(1) $$\begin{array}{c} \text{Degree of substitution}=\frac{162\times A}{1000-\left(58\times A\right)}, \end{array}$$

where *A* is milli-equivalents of consumed acid per gram of sample, 162 is molecular weight of the anhydrous glucose unit, and 58 is the net increase of carboxymethyl group (CH_2_COO^−^).

### 2.6. CMC/Cellulose Hydrogel Preparation

The method for preparation of cellulose hydrogel was performed according to the protocol reported previously [17]. The mixture consisted of 10 ml of NaOH/urea solution (6% (w/v) NaOH/4% (w/v) urea) with 0.4 g of sample and a combination of cellulose and CMC was prepared (as listed in Table 2). The mixture was stirred for 5 min and then stored at −20°C for 12 hours. The frozen solid was thawed at room temperature until the cellulose solution became transparent. The cross-linking reaction was performed by adding 400–1200 *μ*L of epichlorohydrin (ECH) to the sample. The mixture was stirred continuously until it became homogenous and then incubated at 50°C for 2–20 hours. The hydrogel was set, washed with excess water, and lyophilized by freeze-drying at −90°C, 0.018 mBar.

### 2.7. Water Absorbency Determination by Swelling and Reswelling Ratio of Hydrogel

The swelling and reswelling abilities of hydrogel were determined based on the previous study [15]. The dried hydrogel (*W*_*d*_) was weighed and packed in a nylon tea bag and then immersed in excess water for 24 hours. The excess water from the swollen hydrogel in the nylon tea bag was drained, and then the final mass of the hydrogel was determined and considered as *W*_*s*_. The soaked hydrogel was dried in a vacuum oven and immersed into distilled water again to determine the reswelling ratio, where *W*_*r*_ is the weight of reswollen hydrogel. The absorption capacity of hydrogel or swelling ratio was calculated based on the following equation:

(2) $$\begin{array}{c} \text{water absorbency or swelling ratio }\left(\%\right)=\frac{W_{s}-W_{d}}{W_{d}}\times 100, \end{array}$$

(3) $$\begin{array}{c} \text{r}e\text{swelling }\left(\%\right)=\frac{W_{r}-W_{d}}{W_{s}}\times 100. \end{array}$$

### 2.8. Preparation of Cellulose-PEG Composite Using Electrospinning

Cellulose and PEG were dissolved in TFA with the ratio according to Table 3. The procedure was modified from the wok of Ohkawa et al. [24]. The mixture was stirred at 60°C until it became transparent and left for cooling down at room temperature. For WD-MB sample, 3% (w/v) of methylene blue (MB) was added to the mixture and stirred at room temperature. The electrospinning system was set up by loading the polymer solution into a 5 ml syringe that connected to a stainless-steel needle. The positive and negative electrodes were clamped to the needle and copper plate covered with aluminum foils, respectively. The distance between the tip and the collector was set at 15 cm. The fiber was spun on aluminum foil and collected for 3 hours. All samples were prepared according to conditions (as shown in Table 3). Briefly, the DC voltage was applied at 15 kV. The solutions were dispensed at flow rate of 0.5 mL/h under 60% humidity at 25°C. The samples were easily taken from aluminum foil pad by tweezers. The thickness of the nanofiber composite was around 0.04 mm.

### 2.9. Water Absorption Measurement

The method was modified from Tsuge [25]. The dried composite samples obtained from the electrospinning process were cut into 1 cm × 1 cm pieces and weighed (*W*_*d*_). The samples were then immersed into distilled water for 0.5 and 24 hours. After removal of excess water by lint-free paper, the soaked fabrics were weighed (*W*_*t*_). The water absorbency was calculated by equation (2).

### 2.10. Water Vapor Permeability

This method was modified from the work of Zahedi et al [5]. The composite samples were cut into a circle with a diameter of 6 mm and placed on the container with 2 mL of distilled water. The watering-containing container was weighed (*W*_0_) at initial time and placed at 30°C. After 12, 24, and 48 hours, the container was weighed (*W*_*t*_). The water vapor rate was determined according to the following equation:

(4) $$\begin{array}{c} \text{water vapor rate}=\left[\frac{W_{0}-W_{t}}{\pi \times r^{2}t}\right]\times 24, \end{array}$$

where *W*_0_ − *W*_*t*_ is the change in weight due to the water vapor loss (g), *π* × *r*^2^ is the area of exposed film (m^2^), and *t* is the change in time (hour).

### 2.11. Loading Content and Encapsulation Efficiency

The composite samples were weighed (*W*_*S*_) and dissolved in TFA. MB concentration was determined by the absorbance at wavelength of 745 nm using UV spectroscopy. Weight of MB (*W*_MB_) was calculated by linear equation of standard curve. The initial weight of MB (*W*_*i*_) added in the preparation of the composite sample was included. MB loading content and encapsulation efficiency were calculated by the following equation:

(5) $$\begin{array}{c} \text{MB Loading Content }\left(\%\right)=\frac{W_{MB}}{W_{s}}\times 100\%,\\ \text{Encapsulation Efficiency }\left(\%\right)=\frac{W_{MB}}{W_{i}}\times 100\%. \end{array}$$

### 2.12. MB Releasing Test

The composite samples were immersed into distilled water and put in a shaker (175 rpm) at 37°C. The solutions were collected from 1 to 1440 minutes to observe the release of MB by measuring the absorbance at 664 nm by UV spectroscopy. Weight of MB released into the solution (*W*_*r*_) was estimated using the linear equation of calibration curve. The MB releasing content was determined according to the following equation:

(6) $$\begin{array}{c} \text{MB releasing content }\left(\%\right)=\frac{W_{r}}{W_{\text{MB}}}\times 100\%. \end{array}$$

### 2.13. Protein Adhesion

The measurement of BSA protein adsorption on electrospun composite was studied according to the previously reported method using BCA protein assay kit (*Appl. Surf. Sci.* 2016, *386*, 41–50). The 1 cm^2^ of composite samples was immersed in PBS for 24 hours. The samples were then transferred to BSA solution and incubated at 37°C for 3 hours. The surface was washed with PBS before placing it in 3% (w/v) SDS for 30 minutes. The supernatant was collected, and the protein adhesion was determined by measuring the absorbance at 562 nm using a microplate reader (BioTek MQX200 uQuant).

### 2.14. Scanning Electron Microscope (SEM)

The surface and morphology of cellulose fibril, CMC sample, hydrogel, and composite were observed by SEM. The samples were placed on the specimen stub and fixed with conductive carbon double-sided tape. The thickness of each sample was observed under stereomicroscope and then coated with a thin layer of platinum and palladium for 2 min by using an ion sputter (Hitachi E102, Japan) and stored in silica gel until visualization. The surface of the samples was observed and photographed at ×300, ×2000, and ×5000 magnifications with 10 kV accelerating voltage. The image was taken by FE-SEM (Hitachi SU8010 and S4800-I, Japan).

### 2.15. Contact Angle Measurement (CAM)

Dehydrated samples were prepared into a flat shape and then placed on a glass slide. Sessile drop technique was performed by dropping 10–20 *μ*L of distilled water to measure contact angle of cellulose, CMC, and composite samples and then the images were captured by telescope-goniometer and the angle was measured by ImageJ.

### 2.16. Statistical Analysis

GraphPad Prism version 8.3.0 for Windows (GraphPad Software, San Diego, California USA, http://www.graphpad.com/) was used for statistical analysis in this study.

## 3. Results and Discussion

### 3.1. Pretreatment of Cotton Cellulose

**Figure 1:**
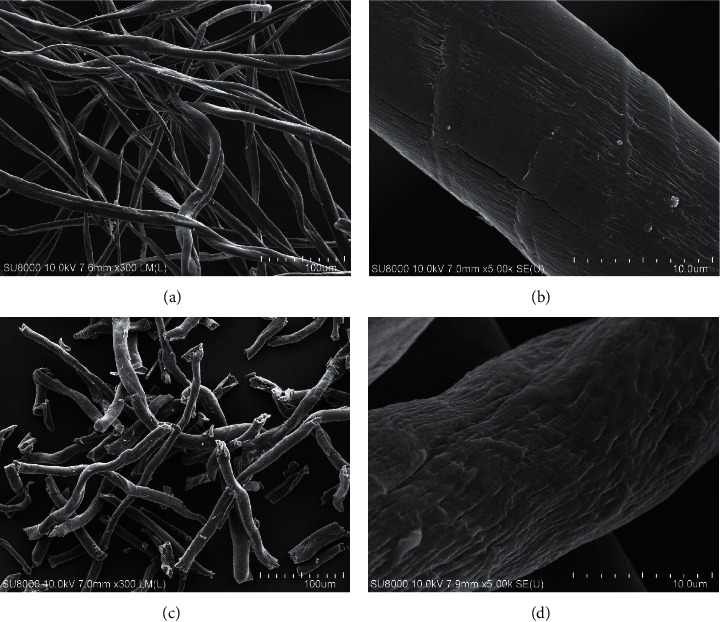
SEM images of raw material surface ((a) and (b)) and pretreated sample ((c) and (d)) under FE-SEM with magnification of 300x and 5000x.

The pretreatment of cotton waste was performed by three steps: alkaline treatment, bleaching, and acid hydrolysis. When compared with the raw material (Figures 1(a) and 1(b)), the surface of fiber after pretreatment (see Figures 1(c) and 1(d)) was rough and shorter in size. However, the diameters of fibers of both samples were similar at around 10 *μ*m. The sign of degradation of fiber appeared after pretreatment. The NaOH solution in the scouring step promoted the dissolution of cellulose allowing removal of impurities including lignin, pectin, waxes, and hemicellulose. The roughness of surface that occurred could lead to an increase in surface area of fiber and enhance the exposure of cellulose to the surface. The bleaching was performed by sodium hypochlorite to remove the colouring matter. The acid hydrolysis was used to enhance the degradation of cellulose providing shorter fibers. The yield of pretreated sample, or cellulose fiber, was 82.5% w/w.

### 3.2. Effect of SMCA on CMC Synthesis

**Figure 2:**
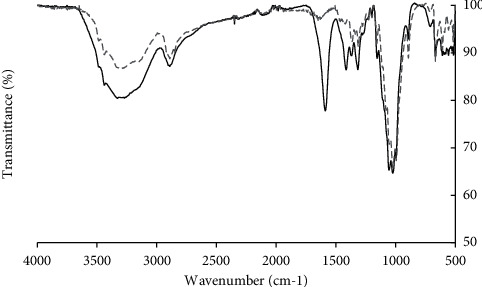
FTIR spectrum of pretreated cellulose (grey dashed line) and CMC 4.8 (black line).

The alkalization step aims to prepare cellulose into appropriate form for etherification; the cellulose fiber was added to isopropanol-water solution followed by dropwise addition of NaOH to produce cellulose anion. Then, the etherification occurred from the interaction of chlorine in SMCA and the cellulose anion at different ratios, resulting in CMC, cellulose with the presence of carboxymethyl ether group on the backbone. The chemical structure and functional group of cellulose and CMC were confirmed by FTIR (see Figure 2). The signal at around 3330–3400 cm^−1^ referred to the stretches of hydroxyl group (−OH) and the intensity of the peak at around 2920 cm^−1^ was observed suggesting the strong signal from –CH- group which was unchanged in CMC and cellulose. The CMC sample was confirmed by the substitution group by the presence of stretching vibration of COO- on its molecule by the strong signal peak at around 1590–1605 (symmetric) and 1415–1420 cm^−1^ (asymmetric). The C-O bonds of ether and alcohol in each glucose unit were detected from the vibration at 1030 cm^−1^ [23, 26].

**Figure 3:**
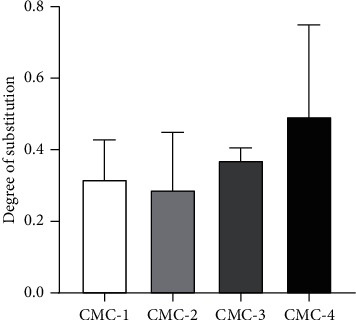
Degree of substitution of CMC. The statistical analysis was done by Tukey's multiple-comparison test. The error bars represent the standard deviation (*n* = 3).

Degree of substitution (DS) is the average amount of substitution of any functional group. In this case, the hydroxyl groups at C2, C3, and C6 carbon atoms are substituted by the carboxymethyl group. The degree of substitution of CMC was determined by the back titration method. CMC was converted into acid form, and then carboxymethyl contents were determined by titration with base. Increase of SMCA led to higher DS. The DS of the products varied from 0.317 to 0.493 (see Figure 3). However, DS of CMC-1 was higher than DS of CMC-2 but there was no significant difference. Therefore, the suitable condition is 20% (w/v) of NaOH and 4.8 g of SMCA (CMC-4) which provided the highest DS at 0.493 ± 0.148. The DS impacts significantly the water interaction property, while DS > 0.4, and the CMC is completely soluble in water. The enhancement of the hydrophilic carboxyl group also helps absorption capacity of hydrogel [15, 27].

**Figure 4:**
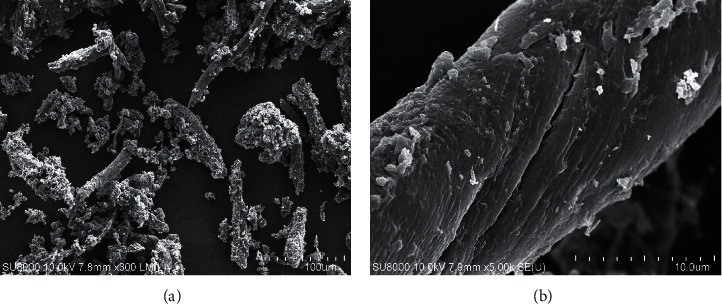
SEM images of CMC sample with magnification of 300x (a) and 5000x (b).

The morphology of CMC was observed under SEM (as shown in Figure 4). When compared with the pretreated sample (Figure 1), an increase in roughness can be clearly observed in the CMC sample. The ruffled surface of CMC samples occurred from additional alkaline treatment steps prior to etherification.

### 3.3. CMC/Cellulose Hydrogel

**Figure 5:**
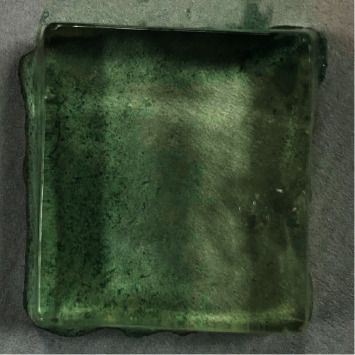
CMC/cellulose hydrogel (Gel-13).

**Figure 6:**
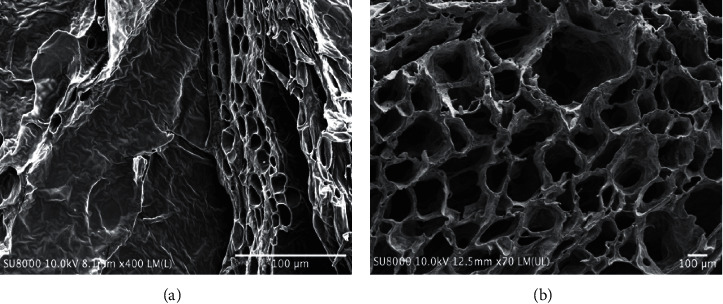
SEM images of cellulose hydrogel with magnification of 400x (a) and CMC/cellulose hydrogel (Gel-13) with magnification of 70x (b).

Hydrogels prepared with different polymer ratios and reaction time were similar in morphology when observed by naked eye (data not shown). The hydrogel was quite transparent and stiff (see Figure 5). In addition, the cross sections of selected hydrogel samples were determined under SEM as displayed in Figure 6. Both samples showed the porous structure; however, the pore sizes were 14.8 ± 3.0 and 215.8 ± 54.1 *μ*m in hydrogel prepared from cellulose and hydrogel prepared from CMC/cellulose, respectively. From the previous study [15], the enlarged porous structure might be resulting from the repulsion of COO- groups of CMC, which could provide additional surface area. This leads to more area for cellulose to interact with water molecules resulting in increase in its ability to hold large amount of water.

### 3.4. Effect of ECH, CMC, and Time on the Water Absorbency

**Figure 7:**
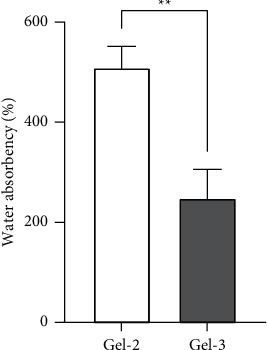
Effect of cross-linker (ECH) on water absorbency. The statistical analysis was performed by unpaired *t*-test. The error bars represent the standard deviation (*n* = 3).

According to the conditions listed in Table 2 and the swelling test shown in Table 4, The solution with cellulose alone (Gel-1) did not form into gel; it remained a clear, colorless liquid after cross-linking reaction. Therefore, the swelling ratio of Gel-1 cannot be determined. This might be due to low concentration of cross-linker which might not be enough to form the rigid structure of hydrogel leading to low interaction with water molecules. Hydrogel samples with higher amounts of ECH showed an increase in water absorption. Cellulose : ECH ratios of 1 : 2 and 1 : 3 (Gel-2 and Gel-3) have swelling ratios at 509 ± 43 and 248 ± 58, respectively (as shown in Figure 7). Larger amount of cross-linker provided low water absorption due to the dense structure which limits the flexibility of water molecules to integrate into hydrogel.

**Figure 8:**
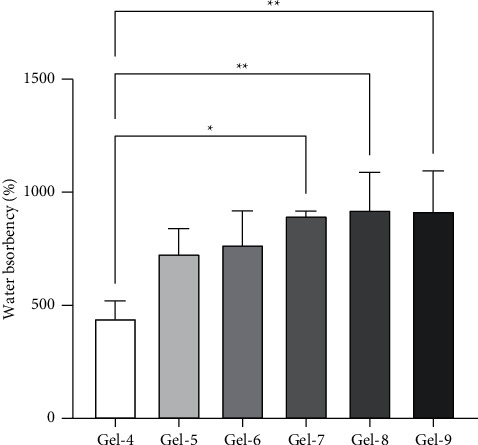
Effect of CMC/cellulose ratio on water absorbency. The error bars represent the standard deviation (*n* = 3). Statistical analysis was performed using one-way ANOVA with Tukey's multiple-comparison test (*p* value: ^*∗*^*p* < 0.05 and ^*∗∗*^*p* < 0.01).

**Figure 9:**
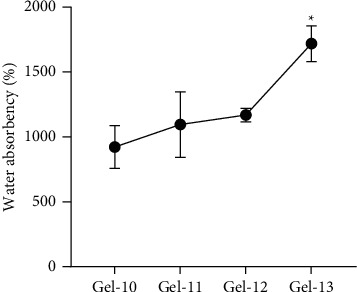
Effect of reaction time on water absorbency. The error bars represent the standard deviation (*n* = 3). All data were compared with 2-hour condition (as control condition) with one-way ANOVA and Dunnett's test (*p* value: ^*∗*^*p* < 0.05).

Increase of cross-linker concentration has been reported to provide a negative effect on the swelling of hydrogel in other materials, such as PEG [28], and superporous hydrogels (SPHs) made from mixed polymers [29]. To promote the water absorption, CMC was added in cellulose-based hydrogel. The presence of CMC could be beneficial for interaction with water, while cellulose was still required to provide support as a backbone structure of hydrogel [30]. Gel-7, Gel-8, and Gel-9 displayed significantly higher water absorption when compared to Gel-4 (*p* value = 0.01, 0.0067, and 0.0074, respectively). Gel-8 and Gel-9 showed the highest swelling ratios at 923 ± 165 and 917 ± 178, respectively (as shown in Figure 8). In addition, the effect of reaction time on the swelling ratio has been investigated. The result shown in Figure 9 suggested that prolonging reaction time can enhance the absorbency. According to this study, hydrogel prepared from CMC/cellulose with 20 hours of reaction time (Gel-13) provided the highest swelling ratio of up to 1718 ± 137, with *p* value <0.05, when compared with control condition (Gel-10). In addition, the rewettability property of hydrogel after immersion in water for 24 hours has been investigated in Gel-13. The reswelling ability was displayed at 97.95 ± 9.76% of initial swollen weight (*W*_*S*_).

### 3.5. Nonwoven Fabrics

**Figure 10:**
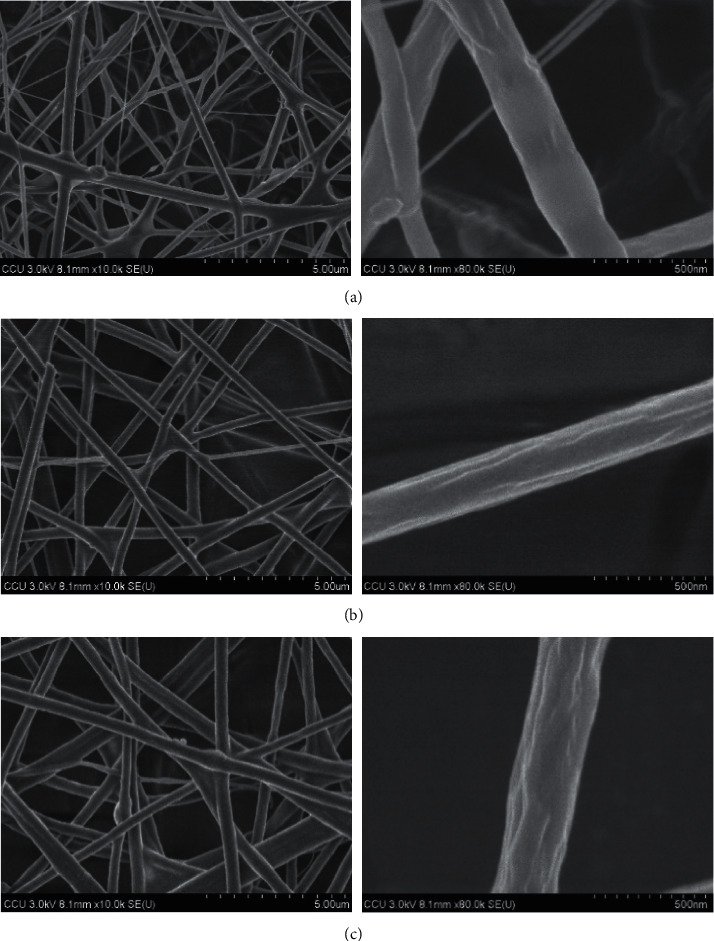
SEM images of polymer solution affected on fiber diameter and surface. (a) 4% (w/v), (b) 6% (w/v), and (c) 8% (w/v) with magnification of 10000x (left) and 80000x (right).

**Figure 11:**
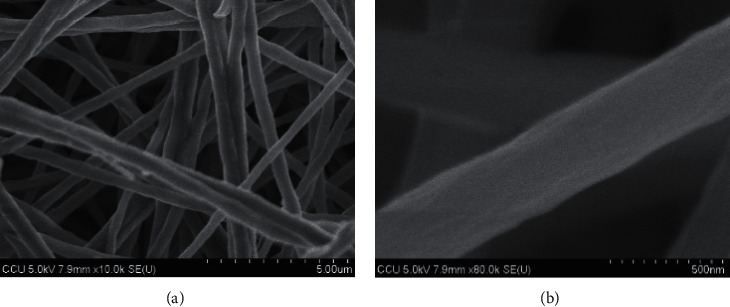
SEM images of effect of 3% (w/v) MB on diameter and surface of fibers with magnification of 10000x (a) and 80000x (b).

**Figure 12:**
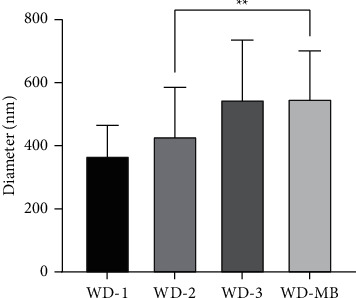
Relationship of average diameter of fiber and polymer concentration and MB. The error bars represent the standard deviation (*n* = 30). Statistical analysis was performed on WD-2 and WD-MB using unpaired *t*-test and Tukey's multiple-comparison test (^*∗∗*^*p* < 0.01).

SEM images are shown in Figure 10. The surface morphology of electrospun composite was prepared from the mixture of PEG and different cellulose concentrations. The smooth surface with uniform diameters and no bead were exhibited with the average diameters of 369 ± 96, 431.2 ± 154.6, and 548 ± 188 nm for 4, 6, and 8% (w/v) of cellulose, respectively. The polymer concentration influences both viscosity and surface tension of solution which acts as an important factor during the electrospinning process. The fiber chain and chain entanglements can be formed at the optimal range of concentration. The fiber may break into droplets before approaching the collector if the polymer concentration or viscosity is too low. Vice versa, the polymer solution will flow difficultly through the needle. Due to the viscosity resistance, the diameter and length of fiber will be higher. WD-2 sample resulted in a smooth surface and less formation of beads [31, 32]. The WD-MB sample of electrospun composite was prepared and the microscopic view was observed under SEM (as shown in Figure 11). The average diameters were 550 ± 151 nm. WD-MB contained the same polymer concentration as the WD-2 sample, but 3% (w/v) of MB was added. The included MB also has some effect on the polymer concentration, viscosity, surface tension, and conductivity of solution. Therefore, the enhancement of these parameters enlarges the diameter of fiber significantly [33] as shown in Figure 12. Then, WD-2 and WD-MB were selected to observe further important properties due to the desirable diameter (50–500 nm approximately) [19].

### 3.6. Water Absorption (WA)

**Figure 13:**
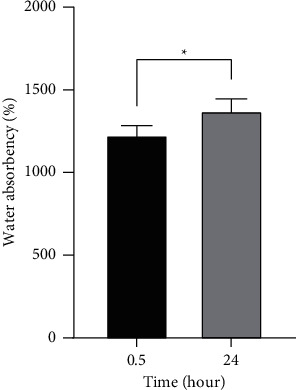
Water absorption of nonwoven fabrics (WD-2). The statistical analysis was done by unpaired *t*-test (^*∗*^*p* < 0.05). The error bars represent the standard deviation (*n* = 3).

Water absorption ratio indicates the hydrophilic properties of electrospinning, fluid absorption, and retention of wound dressing. Cellulose and PEG are both hydrophilic polymers. PEG commonly tends to dissolve in water. However, the PEG acts as minor component to promote the dissolution of cellulose in TFA. The PEG polymer is adulterated inside the electrospun unexposed to the water. In general, wound dressing should provide hydrophilic properties in order to resolve the excessive hydration and reduce swelling and inflammation of the wound [28]. In this experiment, the water absorption of WD-2 (the sample without MB) was investigated. As shown in Figure 13, the sample provided high water absorption of up to 1200% compared with the original weight in the first 30 minutes and remained stable in the aqueous environment after 24 hours with slightly increased water absorption (1300%). Several factors can influence water absorption including the number of water molecules bound to fiber surface, between polymer chains, and held inside a porous structure. Therefore, this material is considered as a promising option as a wound dressing to remove fluid from exudating wounds and prevent undesirable biofouling [34].

### 3.7. Water Vapor Permeability (WVP)

**Figure 14:**
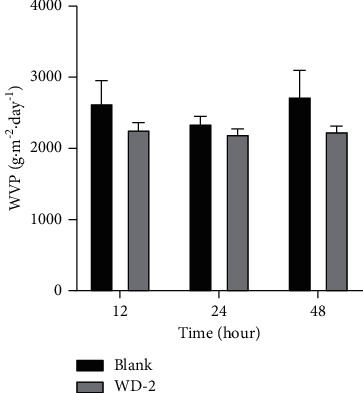
Water vapor permeability rate of nonwoven fabrics. The error bars represent the standard deviation (*n* = 3).

When compared with control samples, the results showed that moisture can penetrate through WVP of composite to surrounding by 2285.92 ± 44.2, 2285.92 ± 37.45, and 2163.84 ± 23.14 g/m^2^·day^−1^ after 12, 24, and 24 hours, respectively (as shown in Figure 14). The suitable range of WVP has been suggested at about 2000–2500 g·m^−2^day^−1^ as it could reduce water and moisture lost to the atmosphere in the previous report [5]. The suitable WVP rate promotes the wound dehydration and scar generation. In contrast, low WVP rate can bring about exudate accumulation at wound, resulting in delaying healing process and occurring infection [35, 36].

### 3.8. Wetting Properties

The suitable wound dressing should have high wettability which refers to the hydrophilic properties and the ability to absorb the wound exudate and maintain moisture content. These qualifications are indicated by the angle under 90° [37]. The enlargement of contact angle can be affected by increased rough surface and hydrophobicity of composites which restrain the spread of water [38]. The contact angle of WD-2 in this study was at 34.5 ± 7.4°. However, contact angle of WD-1,3 and MB cannot be measured since the water droplets immediately dispersed when contacted to the fiber. It indicated strong hydrophilic properties that may decrease the fiber stability [39]. Therefore, the ratios of polymer and MB or antibacterial should be adjusted to reduce the wetting properties to the suitable level.

### 3.9. Loading Content, Encapsulation Efficiency, and MB Releasing

**Figure 15:**
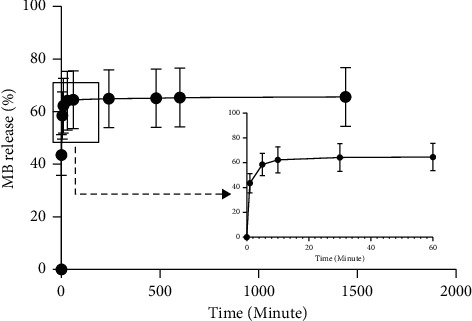
MB releasing profile at 1440 minutes. The error bars represent the standard deviation (*n* = 3).

The amounts of MB loaded in cellulose/PEG electrospinning samples were influenced by the structure and physical and chemical properties. High encapsulation efficiency may not be correlated with the loading content. The loading content also depends on the structure and physical and chemical properties of electrospun composite [40]. The WD-MB sample showed the average encapsulation efficiency was 55.76 ± 17.89%, and loading content was 15.21 ± 4.88%. MB loaded in WD-MB sample was released into distilled water at room temperature.  Figure 15 presents 64.6% of MB discharged within 1 hour and then discharged gradually until 65.7% of loaded MB at 24 hours. It suggested that the MB releasing profile of WD-MB was characterized as an immediate MB release due to the quick dispersion of MB after taking part in the target environment for an hour [41]. Generally, encapsulation techniques, types of loaded MB, and electrospun polymers are engaged in release behavior. In this study, MB was mixed simply with polymer solution (blending method). Loaded MB is located at or near the fiber surface; thus it rapidly blasts into the aqueous environment within an hour [21].

### 3.10. Protein Adsorption

The desirable wound dressing should be nonadherent to the wound and easy to remove after the healing process [6]. The BSA is represented as a protein model on the skin and wound; therefore, the amount of BSA distributed into the SDS should be as low as possible. The result showed that only 0.6943 ± 0.16 *μ*g/cm^2^ of BSA was adhered on both surfaces of 1 cm^2^ WD-2 sample immersed in 2 mL of 2 mg/mL of BSA. Protein adsorption studies were also compared to other materials. The results from Nasreen et al. [42] show the highest BSA adsorption on PVDF-HEMA electrospun membrane around 0.49 ug/cm^2^.

## 4. Conclusion

In this study, we successfully purified cotton waste from textile company into cellulose and modified it as CMC with desirable DS. Both cellulose and CMC have become our crucial materials for cellulose-based hydrogel and nonwoven composite. CMC/cellulose hydrogel achieved by chemical cross-linking had high swelling and reswelling ability. Cellulose/PEG composites were successfully prepared by electrospinning technique and exhibited the smooth surfaces with desirable diameter. The nanocomposite also had high fluid absorption and moisture retention in suitable range and was nonadherent to protein. According to the results, the suitable exudate absorption, wound dehydration, and tissue regeneration environment were successfully provided. Electrospun sample with MB loaded has demonstrated acceptable encapsulation efficiency and loading content and offered an immediate MB release profile. According to their properties, the hydrogel and nonwoven fabrics developed in this work could be potential materials for development of wound dressing. However, further studies including biocompatibility, cytotoxicity to epithelial cells, gas transmission, mechanical stability, and antibacterial photodynamic activity of nanofiber composite must be investigated to cover requirements of wound dressing substance.

## Figures and Tables

**Table 1:** Chemical compositions and reactive time condition of CMC.

Code	SMCA (g)
CMC-1	1.2
CMC-2	2.4
CMC-3	3.6
CMC-4	4.8

**Table 2:** Chemical compositions and reactive condition of hydrogel.

Code	Cellulose	CMC	ECH (mL)	Time (h)
Gel-1	0.4	0	0.4	2
Gel-2	0.4	0	0.8	2
Gel-3	0.4	0	1.2	2
Gel-4	0	0.4	0.8	2
Gel-5	0.2	0.2	0.8	2
Gel-6	0.16	0.24	0.8	2
Gel-7	0.12	0.28	0.8	2
Gel-8	0.08	0.32	0.8	2
Gel-9	0.04	0.36	0.8	2
Gel-10	0.08	0.32	0.8	2
Gel-11	0.08	0.32	0.8	6
Gel-12	0.08	0.32	0.8	15
Gel-13	0.08	0.32	0.8	20

**Table 3:** Polymer composition and synthesis conditions of nonwoven fabrics.

Code	Cellulose % (w/v)	PEG % (w/v)	MB conc.% (w/v)
WD-1	4	2	0
WD-2	6	2	0
WD-3	8	2	0
WD-MB	6	2	3

**Table 4:** Swelling ratio of hydrogels after immersing in distilled water for 24 hours.

Code	Swelling ratio (%)
Gel-1	—
Gel-2	509 ± 43
Gel-3	248 ± 58
Gel-4	443 ± 77
Gel-5	729 ± 110
Gel-6	769 ± 150
Gel-7	897 ± 21
Gel-8	923 ± 165
Gel-9	917 ± 178
Gel-10	923 ± 165
Gel-11	1095 ± 252
Gel-12	1169 ± 53
Gel-13	1718 ± 137

## Data Availability

All data created during this research are available from the corresponding author upon request.

## Supplementary Materials

Supplementary Materials Figure S1 and Table S1: standard curve and linear equation for determining MB loading content and encapsulation efficiency. Figure S2 and Table S2: standard curve and linear equation for determining MB releasing content.
