# Supplementary material for: Cotton Cellulose-Derived Hydrogel and Electrospun Fiber as Alternative Material for Wound Dressing Application
Source: Int J Biomater. 2022 Mar 7;2022:2502658. doi: 10.1155/2022/2502658 (PMC8920707; doi:10.1155/2022/2502658)
Supplement: Supplementary Materials — Figure S1 and Table S1: standard curve and linear equation for determining MB loading content and encapsulation efficiency. Figure S2 and Table S2: standard curve and linear equation for determining MB releasing content. [file 2502658.f1.docx]

# Supplementary information

Table S1: Absorbance according to methylene blue concentration for determining MB loading content and encapsulation efficiency.

| **MB concentration (mg/ml)** | **Absorbance** |
| --- | --- |
| 0 | 0 |
| 7.72475E-05 | 0.019 |
| 0.000102995 | 0.028 |
| 0.000137329 | 0.034 |
| 0.000183106 | 0.049 |
| 0.000244141 | 0.065 |
| 0.000341797 | 0.098 |

FigureS1: Standard curve and linear equation for determining MB loading content and encapsulation efficiency.

Table S2: Absorbance according to methylene blue concentration for determining MB releasing content.

| **MB concentration (mg/ml)** | **Absorbance** |
| --- | --- |
| 0 | 0 |
| 7.72E-05 | 0.021 |
| 8.61E-05 | 0.025 |
| 0.000103364 | 0.031 |
| 0.000124036 | 0.035 |
| 0.000148844 | 0.041 |
| 0.000178612 | 0.050 |
| 0.000214335 | 0.057 |
| 0.000257202 | 0.068 |
| 0.000308642 | 0.081 |

Figure S2: Standard curve and linear equation for determining MB releasing content
